# Do Leg-Focused Exercises Improve Arm and Hand Function for Individuals With Neurologic Disorders? A Scoping Review

**DOI:** 10.1016/j.arrct.2025.100572

**Published:** 2026-01-21

**Authors:** Katrina S. Nietsch, Lauren E. Kinne, Gloria Willson, Noam Y. Harel, Lynda M. Murray

**Affiliations:** a Department of Rehabilitation and Human Performance, Icahn School of Medicine at Mount Sinai, New York, NY; b Spinal Cord Damage Research Center, James J. Peters VA Medical Center, Bronx, NY; c Levy Library, Icahn School of Medicine at Mount Sinai, New York, NY; d Department of Neurology, Icahn School of Medicine at Mount Sinai, New York, NY

**Keywords:** Central pattern generators, Hand function, Leg exercise, Leg training, Neural coupling, Neurologic disorders, Recovery of function

## Abstract

**Objective:**

To perform a scoping review summarizing existing literature on the use of leg exercises to improve arm function for individuals living with neurologic conditions.

**Data Sources:**

We searched 5 databases (PubMed, Embase, Cumulative Index to Nursing and Allied Health Literature, Scopus, Cochrane Central), secondary references, and 9 gray literature databases from January 1, 1947, to October 28, 2025.

**Study Selection:**

Studies were included if they involved adults living with neurologic conditions conducting leg-focused exercise programs that included an arm-specific outcome. All were written in English. Two reviewers independently screened all articles.

**Data Extraction:**

Data were extracted by one reviewer and confirmed by a second. Methodological quality was assessed using Physiotherapy Evidence Database scale.

**Data Synthesis:**

A total of 12 published studies met criteria. Studies involved 6-101 participants living with deficits because of stroke or Parkinsons disease. Participants performed leg-focused exercises, ranging from a single session up to 100 sessions, followed by upper extremity assessments. Of the 12 studies, 6 showed a statistically significant or clinically relevant benefit of leg exercise on arm function. However, these were mostly observed as incidental findings.

**Conclusions:**

This scoping review highlights a gap in research on the use of leg training to activate arm motor circuits for individuals living with neurologic conditions. Future research should explore this approach as a novel method for neurorehabilitation.

## Highlights

• Critical gap in understanding the therapeutic impact of leg exercises on arm function.
• Leg training interventions show potential for arm functional recovery.
• Future research should explore leg training modes and mechanisms for arm rehabilitation.

Neurologic disorders such as stroke, Parkinson disease (PD), spinal cord injury (SCI), and amyotrophic lateral sclerosis (ALS) often impair upper extremity function, causing chronic impairments in activities of daily living (ADLs). Exploring effective upper extremity rehabilitation strategies is crucial to improving ADLs and transitioning to independent living.

To date, more emphasis in stroke, PD, SCI, and ALS rehabilitation has been placed on lower rather than upper extremity therapy. Early animal work identified autonomous neural networks in the spinal cord, termed central pattern generators (CPGs), which are responsible for rhythmic behaviors such as locomotion.^1^, ^2^, ^3^, ^4^, ^5^, ^6^, ^7^ Further advances in quadruped animal models have revealed evidence for anatomically and functionally distinct cervical and lumbar CPGs for forelimbs and hindlimbs, which can be easily coupled or uncoupled depending on the task.^8^ These circuits cooperatively integrate peripheral sensory, propriospinal, and descending supraspinal feedback mechanisms, ensuring effective task-dependent coordination of all four limbs during rhythmic activities.^4^^,^^9^, ^10^, ^11^, ^12^

Humans, although bipedal, retain evidence of inter-CPG communication between lumbar and cervical circuits, demonstrated during postural^13^ and ambulatory perturbations,^14^ recumbent leg stepping,^15^^,^^16^ and with arm swing during normal gait.^17^ The interlimb neural coupling is also highlighted by bidirectional reflex responses of the arms and legs, in which leg muscle Hoffmann-reflexes are modulated by arm position and vice versa.^18^

Rehabilitation approaches have successfully leveraged inter-CPG communication, showing that arm swing or hand cycling can improve leg and gait function in individuals with neurologic conditions.^19^^,^^20^ However, little research has focused on the reverse approach, using leg exercises to improve arm function, despite evidence that leg training can induce global experience-dependent plasticity,^21^ and promote corticospinal excitability^22^ in arm and hand muscles. Therefore, this scoping review aims to identify and describe studies of leg training with arm and hand outcomes in individuals with neurologic conditions by answering “Do leg-focused exercises improve arm and hand function for individuals with neurologic disorders?” The novelty of this topic necessitated a broad review to identify disparate studies with varying objectives, patient populations, and designs. Therefore, we determined that a scoping review was appropriate to address the research question.

## Methods

### Review methodology

The review followed the Joanna Briggs Institute (JBI) Scoping Review Methodology Groups guidelines^23^ and the Preferred Reporting Item for Systematic Reviews and Meta-Analysis for Scoping Review guidelines and framework.^24^ The review was not preregistered.

### Research question

The research question “Do leg-focused exercises improve arm and hand function for individuals with neurologic disorders?” was developed using the Population/Concept/Context framework.^23^

### Selection process

The JBI and Preferred Reporting Item for Systematic Reviews and Meta-Analysis for Scoping Review frameworks also informed the inclusion and exclusion criteria (table 1). On the basis of our preliminary search of relevant keywords, it was evident that we needed to increase the date range and number of databases searched. Because of limited resources, the search was restricted to English. Studies were excluded because of unclear interventions, or the inclusion of whole-body exercises as an intervention aside from those performed as part of inpatient standard of care, and where upper extremity outcome measures were not reported separately as part of a functional assessment (eg, International Standards for Neurological Classification of Spinal Cord Injury [Impairment Scale], Functional Independence Measure, Unified Parkinsons Disease Rating Scale [UPDRS]).

**Table 1:** Study inclusion and exclusion criteria.

Inclusion Criteria	Exclusion Criteria
Type of publications	

Peer-reviewed articles	Study protocols, brochures, guidelines, patents, book chapter reports

Original research, primary studies	

Gray literature: conference abstracts, commentaries, essays, consensus statements, posted clinical trial results, theses	

Published date	

January 1, 1947, to October 28, 2025	Any date outside this range

Design	

Randomized and nonrandomized controlled studies, case study/report, observational, case series, quasi-experiment, pre/post tests	Reviews

Language	

English or foreign languages translated into English	Any other language

Population	

Adults (>18 y old) living with neurologic disorders	People <18 y old, or any other diagnosis

Studies with participation from any country	Studies without a clear definition of adult participants living with neurologic disorders

Topic of interest/intervention	

Studies presenting results from individuals living with neurologic disorders or injuries	Studies addressing issues other than research or therapy involvement of individuals with neurologic disorders

Interventions implementing leg-focused training (ie, range of motion, sit-to-stand, standing, walking, jumping, kicking, and/or strength training)	Cognitive and motor dual-task training

	Any other type of intervention

Outcome	

Any neurobiological technique addressing upper extremity function, neurorehabilitation, or neurophysiology	Any other technique or test subset where the outcome of the upper extremity assessment is not clearly distinguished

### Search strategy

Author 1 (A1), Author 4 (A4), and Author 5 (A5) performed an initial citation search across multiple databases using citation mining, forward/backward citation searching, and author/group citation search strategies to narrow down the most appropriate terms used in the literature.

A medical librarian (Author 3, A3) performed comprehensive searches on November 29, 2023, and October 28, 2025, within five databases: PubMed, Embase (Ovid), Cumulative Index to Nursing and Allied Health Literature, Scopus, and Cochrane Central, including a gray literature search on November 29, 2023, within nine databases: Google Scholar (first 5 pages), medRxiv, Australia New Zealand Clinical Trials Registry, European Union Clinical Trials Registry, US government web-based resource ClinicalTrials.gov, ProQuest Dissertation/Theses, World Health Organization (WHO) Library, WHO Global Index Medicus, and WHO International Clinical Trials Registry Platform. The searches used proximity, adjacency, and/or exploded search strategies (a built-in database thesaurus for nested words), which concluded on November 18, 2025 (table 2, supplemental table S1, available online only at http://www.archives-pmr.org/).

**Table 2:** Search strategy.

Peer-reviewed sources: PubMed Return date range: January 1, 1947 to October 28, 2025 Using proximity/adjacency search between 4 and 8 keywords and unlimited truncation of selective words in a citation’s title, collection title, abstract, other abstract, and author keywords using the [tiab] tag and asterisk
**PubMed Query [1102 results]** Modified search: ((leg[tiab] OR "leg exercise"[Title/Abstract:∼5] OR "leg cycling"[Title/Abstract:∼5] OR "leg walking"[Title/Abstract:∼8] OR "leg running"[Title/Abstract:∼5] OR "leg movement"[Title/Abstract:∼5] OR "leg sport"[Title/Abstract:∼5] OR "leg strength"[Title/Abstract:∼5] OR "leg activity"[Title/Abstract:∼5])) AND (((Neurological Rehabilitation) OR "Nervous System Diseases"[Mesh] OR "spinal injuries"[Title/Abstract:∼5] OR "spinal injury"[Title/Abstract:∼5] OR neurolog*[tiab] OR spinal[tiab]) AND ((hand[tiab] OR "hand strength"[Title/Abstract:∼5] OR arm[tiab] OR "upper extremity"[Title/Abstract:∼4] OR "upper limbs"[Title/Abstract:∼4]) AND ((Function[tiab] OR Recover*[tiab] OR Strength[tiab] OR Dexter*[tiab] OR Grip[tiab] OR "Recovery of Function"[Mesh]))))

NOTE. The complete search strategies for all peer-reviewed sources, including grey literature and secondary references, are detailed in supplemental table S1.

Author 2 (A2) and A5 hand-searched the reference lists of studies that met the inclusion criteria to identify additional records.

### Information source handling

All peer-reviewed database sources were downloaded into Covidence, a reference management program.^25^ Covidence handled duplications, visually inspected by A5. Following JBI guidance, a sample of 25 titles/abstracts was independently reviewed by A1, A2, A4, and A5. Reaching 88% agreement on the sample of 25 titles/abstracts, discrepancies were then discussed, and criteria were refined in table 1 for clarity.^23^ Two reviewers (A1, A2, or A5) assessed each remaining article, with a third reviewer (A4) resolving conflicts. Inaccessible articles were excluded. The same workflow was applied to gray literature and secondary references.

### Data charting

Key information was extracted into a charting table by one reviewer (A5) and confirmed by a second (A4): author(s), publication year, title, hypothesis, participant demographics, methodology, intervention type/duration, outcome measures, and key findings related to the research question. Methodological quality was evaluated using the Physiotherapy Evidence Database scale,^26^ with classifications as “poor” (0-3), “fair” (4-5), “good” (6-8), or “excellent” (9-10).

## Results

### Study search and selection

**Fig 1:**
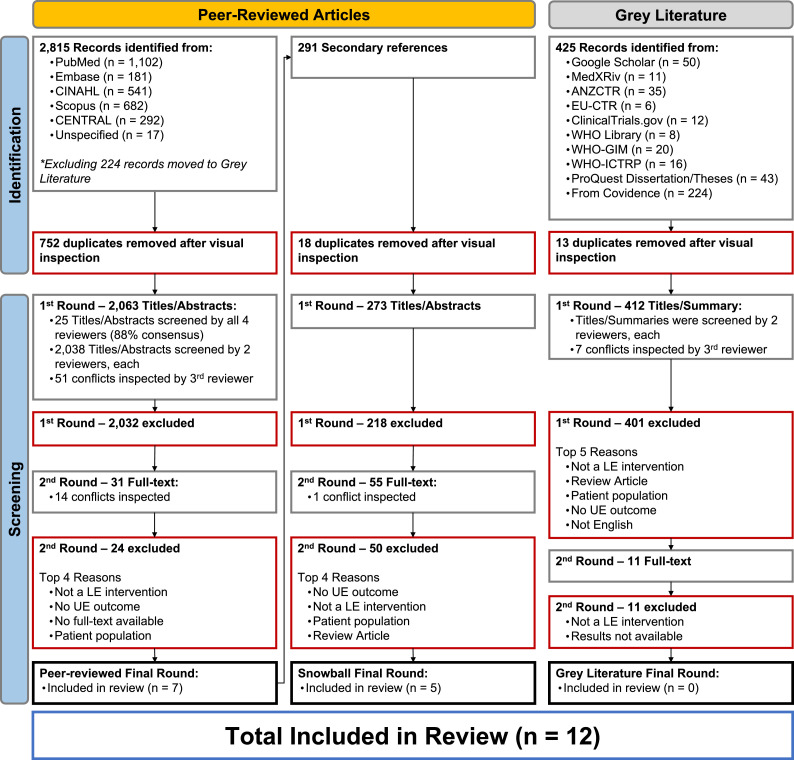
PRISMA flowchart. Peer-reviewed literature searches were conducted across 5 databases: PubMed, Embase (Ovid), Cumulative Index to Nursing and Allied Health Literature (CINAHL), Scopus, and Cochrane Central. A gray literature search was conducted in Google Scholar, medRxiv, Australia New Zealand Clinical Trials Registry (ANZCTR), European Union Clinical Trials Registry (EU-CTR), US government web-based resource ClinicalTrials.gov, ProQuest Dissertation/Theses, World Health Organization (WHO) Library, WHO Global Index Medicus (WHO-GIM), and WHO International Clinical Trials Registry Platform (WHO-ICTRP). LE, lower extremity; UE, upper extremity.

The search identified 3531 records (fig 1), with 2748 remaining after duplicate removal. After title and abstract screening, 2651 were excluded, leaving 97 for full-text review. Twelve peer-reviewed publications met inclusion criteria. Data from the 12 published studies are summarized in table 3. Common reasons for exclusion were the absence of leg-focused interventions; unclear upper extremity outcomes; or failure to meet other criteria.

**Table 3:** Data extraction from included peer-reviewed publications.

Study Article (By Date)	Hypothesize That LE Exercise Would Improve UE Outcomes?	Study Design and Methodology	Study Population (Mean±SD) or Mean (Range)	PEDro Score	LE Exercise Influence on UE Metrics	Summary of Key UE Findings
Hesse et al (1995)^27^	No	**Single-group crossover:** A-B-A **Training intervention:** (A) partial BWSTT; (B) Bobath approach PT **Study duration:** 15 sessions (30 min for BWSTT; 45 min for Bobath) over 3 wk for each phase **Assessment timepoints:** walking performance twice a week, all other outcomes tested weekly **Outcome measures:** FAC, MAS (wrist, ankle), Motricity Index (upper, lower); Rivermead Motor Assessment, walking ability	**Population:** stroke, moderate to severe **Sample size:** n=7 (6M,1F) **Age:** 60.3±8.7 y **TSI/D:** Chronic; 181.6±106.2 d	Not scored	=	No consistent changes in muscle strength or tone were observed after either partial BWSTT or Bobath approach PT. **MAS** (wrist) (mean±SD): A-B-A: baseline 2.4±1.5, A1 (wk 3) 2.1±1.1, B (wk 3) 2.9±0.6, A2 (week 3) 2.7±0.7 **Motricity Index** (arm) (mean±SD): A-B-A: baseline 18.0±16.7, A1 (wk 3) 18.7±17.7, B (wk 3) 20.0±19.7, A2 (week 3) 20.0±19.7

Kwakkel et al (1999)^28^	No	RCT: 3 parallel groups Training intervention: Leg group: sitting, standing, weight-bearing, treadmill training if available; Arm group: leaning, punching a ball, grasping, and moving objects; Immobilization: inflatable pressure splitting of paretic arm and leg while lying supine Study duration: 100 sessions (30 min) over 20 wk Assessment timepoints: wk 0, 6, 12, 20, and 26 Outcome measures: Primary: ADL, walking ability, ARAT (paretic arm). Secondary: 10MWT, Nottingham Health Profile (Part 1 only), Sickness Impact Profile (short 68-item version), Frenchay Activities Index	Population: stroke, severe Leg group Sample size: n=31 (13M,18F) Age: 64.5±9.7 y TSI/D: acute to early-subacute; 7±2.5 d Arm group Sample size: n=33 (16M,17F) Age: 69±9.8 y TSI/D: acute to early-subacute; 7.2±2.8 d Control group Sample size: n=37 (14M,23F) Age: 64.1±15 y TSI/D: acute to early-subacute; 7.5±2.9 d	7	+	Arm and leg training led to significant improvements in dexterity of the paretic arm. All time points for the leg group were significantly better than the control group (*P*<.01). In addition, week 12 (*P*<.05), 20 (*P*<.01), and 26 (*P*<.05) in the arm group were significantly better than the control group. However, no significant difference was seen between training groups at any timepoint. ARAT (median [IQR]): baseline, wk 6, wk 12, wk 20, wk 26 Leg group: 0 [0-6]), 1 [0-43], 2 [0-53], 2 [0-56], 3 [0-56] Arm group: 0 [0-1], 1 [0-14], 3 [0-34], 9 [0-39], 4 [0-38] Control group: 0 [0-0]), 0 [0-1], 0 [0-1], 0 [0-2], 0 [0-2.25]

Dean et al (2000)^29^	No	RCT: 2 groups Training intervention: Leg group: progressive circuit program (5 min per station). Arm group: strengthening, dexterity, grasp and releases, ROM Study duration: 12 sessions (60 min) over 4 wk Assessment timepoints: week 0, 4, 12 Outcome measures: 10MWT, 6MWT, TUG, step test, STS, walking quality, grip strength, Purdue peg board	Population: stroke, mild Leg group Sample size: n=5 (2M,3F) Age: 68.8±4.7 y TSI/D: Chronic; 2.1±0.5 y Arm group Sample size: n=4 (3M,1F) Age: 64.8±3.3 y TSI/D: Chronic; 1.7±0.9 y	5	=	No significant between group differences in grip strength or hand dexterity were found. Grip strength (kg) (mean±SD): baseline, wk 4, wk 12 Leg group: 21.2±11.0, 20.1±13.1, 20.5±16.7 Arm group: 13.3±11.4, 15.7±11.0, 15.2±13.8 Purdue Peg board (unimanual) (mean±SD): baseline, wk 4, wk 12 Leg group: 4.6±3.5, 5.2±3.4, 4.5±3.3 Arm group: 3.3±3.8, 4.8±5.6, 4.8±5.5 Purdue Peg board (bimanual, pairs) (mean±SD): baseline, wk 4, wk 12 Leg group: 3.8±2.9, 4.0±2.6, 3.0±2.2 Arm group: 2.8±3.2, 4.0±4.6, 3.3±3.8

Bourbonnais et al (2002)^30^	No	RCT: 2 groups Training intervention: Leg group: force training of the paretic limb. Arm group: force training of the paretic limb Study duration: 18 sessions (60 min) over 6 wk Assessment timepoints: wk 0, 6, 14 Outcome measures: 12-meter self-paced walking velocity, 2MWT, TUG, MAS, FMA, TEMPA, BBT, finger-to-nose test, pain	Population: stroke, moderate Leg group Sample size: n=12 (6M,6F) Age: 44.6±14.1 y TSI/D: chronic; 34.7±16.1 mo Arm group Sample size: n=13 (10M,3F) Age: 47.2±13.9 y TSI/D: chronic; 37.3±14.3 mon	5	+	Both leg and arm training groups demonstrated similar improvements for FMA (F=4.21, *P=*.021), and finger-to-nose (F=4.43, *P=*.026), and box and block (F=3.86, *P=*.04) assessments over time, although changes were not considered clinically significant. FMA (mean±SD): baseline, wk 6, wk 14 Leg group: 40.6±17.3, 41.8±17.3, 40.3±18.0 Arm group: 43.4±15.3, 44.9±16.1, 43.0±14.8 Finger-to-nose (mean±SD): baseline, wk 6, wk 14 Leg group: 7.8±7.1, 9.3±9.7, 9.9±10.3 Arm group: 10.3±7.8, 11.6±8.6, 11.4±8.4 BBT (mean±SD): baseline, wk 6, wk 14 Leg group: 22.8±20.8, 25.5±25.2, 24.9±24.8 Arm group: 21.8±16.8, 24.3±18.8, 24.7±21.3

Blennerhassett and Dite, (2004)^31^	No	RCT: 2 groups Training intervention: 10 circuit stations (5 min each). Leg group: cycling endurance, walking endurance, sitting, standing, step-ups, obstacle course walking, standing balance, stretching, strengthening. Arm group: arm ergometer, reach and grasp, hand-eye coordination, stretching, strengthening Study duration: 20 sessions (60 min) over 4 wk Assessment timepoints: wk 0, 4, 28 Outcome measures: TUG, step test, 6MWT, JTHFT, MAS	Population: stroke, moderate Leg group Sample size: n=15 (8M,7F) Age: 53.9±19.8 y TSI/D: early-late subacute; 36±25.1 d Arm group Sample size: n=15 (9M,6F) Age: 56.3±10.5 y TSI/D: early-late subacute; 50.1±49.2 d	8	=	No significant improvement in the JTHFT or UE MAS was observed in the leg training group. JTHFT (s) (mean±SD): baseline, wk 4, mo 6 Leg group: 39.6±32.7, 30.8±19.9, 31.0±33.2 Arm group: 41.8±19.4, 21.3±11.3 (*P*=.005, baseline), 23.6±12.2 (*P*=.005, baseline) MAS (upper arm) (median [IQR]): baseline, wk 4, mo 6 Leg group: 5 (1-6), 6 (4-6), 6 (5-6) Arm group: 5 (2-5), 6 (5-6) (*P*=.001, baseline), 5 (4.5-6) (*P*=.004, baseline) MAS (hand) (median [IQR]): baseline, wk 4, mo 6 Leg group: 6 (2-6), 6 (5-6), 6 (3-6) Arm group: 6 (0-5), 6 (5-6), 5 (4.2-6)

Mehrholz et al (2006)^32^	No	Case series Training intervention: 3 assisted jumping exercises (repetitive jump, high jump, and long jump) Study duration: 30 sessions (5-7 min) over 6 wk Assessment timepoints: week 0 and 6 Outcome measures: Motricity Index, FMA, MTS, FAC, 10MWT, 6MWT, stride length, Rivermead Visual Gait Index	Population: stroke, mild to moderate Sample size: n=6 (5M,1F) Age: 54±9.2 y TSI/D: early-late subacute; 6.7±3.3 wk	Not scored	=	UE measures for the MTS and FMA remained unchanged over time; no impact from leg jump training. FMA (upper limb): baseline 39.5±2.1, wk 6 39.2±2.0 (*P*=.157)

Pang et al (2006)^33^	No	RCT: 2 groups Training intervention: Leg group: brisk walking, sitting, standing, alternate step-ups, partial squats, toe rises, mobility and balance training. Arm group: shoulder TheraBand exercises, passive/active range of motion, weight-bearing activities (eg, pushing down of a physio ball, push-ups on armrest), dumbbell/wrist cuff weight exercises, Thera putty exercises, playing cards, picking up objects, reaching tasks, fine motor tasks, and electrical stimulation of wrist extensors (only for those with less than 20 degrees of active wrist extension) Study duration: 57 sessions (60 min) over 19 wk Assessment timepoints: week 0 and 19 Outcome measures: Primary: WMFT, FMA. Secondary: grip strength, MAL	Population: stroke, mixed severity Leg group Sample size: n=30 (18M,12F) Age: 66±8.7 y TSI/D: chronic; 5.2±5.0 y Arm group Sample size: n=30 (18M,12F) Age: 64.9±8.5 y TSI/D: chronic; 5.1±3.6 y	7	=	Leg group showed little to no significant change in WMFT, FMA, or grip strength. WMFT (functional ability) (mean±SD) (between group, *P*=.003) Leg group: baseline 3.7±1.5, wk 19 3.7±1.5 Arm group: baseline 2.9±1.6, wk 19 3.1±1.8 WMFT (task 8-15 only) (mean±SD) (between group, *P*=.011) Leg group: baseline 3.5±1.6, wk 19 3.5±1.8 Arm group: baseline 2.6±1.7, wk 19 2.9±1.9 WMFT (s) (mean±SD) (between group, *P*=.177) Leg group: baseline 2.5.5±48.4, wk 19 25.3±48.2 Arm group: baseline 29.7±47.1, wk 19 26.9±47.5 FMA (mean±SD) (between group, *P*=.001) Leg group: baseline 51.3±19.2, wk 19 52.5±18.5 Arm group: baseline 40.8±19.6, wk 19 45.7±19.3 Grip strength (N) (mean±SD) (between group, *P*=.203) Leg group: baseline 166.6±128.8, wk 19 170.7±131.6 Arm group: baseline 97.3±94.8, wk 19 113.8±96.7 MAL (mean±SD) (between group, *P*=.274) Leg group: baseline 3.0±1.7, wk 19 3.3±1.7 Arm group: baseline 1.7±1.5, wk 19 2.3±1.7

Fisher et al (2008)^34^	No	RCT: 3 groups Training intervention: BWSTT group: progressive moderate-high-intensity (>3 METs) BWSTT. PT group: low-moderate multimodal exercises (1-3 METs). Control group: 6 passive educational sessions Study duration: 24 sessions (45 min) over 8 wk (Control: 6 educational sessions (60 min) over 8 wk) Assessment timepoints: week 0 and 8 Outcome measures: UPDRS-III, 10MWT (maximum speed vs self-paced), STS, TMS-evoked CSP of the FDI muscle	Population: PD, mild to moderate BWSTT group Sample size: n=10 (6M,4F) Age: 64.0 ± 14.5 y TSI/D: 14.7 ± 9.9 mo PT group Sample size: n=10 (5M,5F) Age: 61.5 ± 9.8 y TSI/D: 8.8 ± 7.9 mo Control group Sample size: n=10 (8M,2F) Age: 63.1 ± 11.5 y TSI/D: 17.7 ± 13.3 mo	5	˄	High-intensity leg training increased FDI CSP duration (more affected hemisphere baseline 175.4±35.3, wk 8 207.3±27.1; mean±SD), in contrast to a mean decrease or no change in the low or no-exercise groups. CSP (average change) BWSTT (high): more affected hemisphere 32 ms increase; less affected hemisphere 19.4 ms increase PT (low): more affected hemisphere 17 ms decrease; less affected hemisphere no change Control: more affected hemisphere no change; less affected hemisphere 6.5 ms decrease

Ridgel et al (2009)^35^	No	RCT: 2 groups Training intervention: FE group: assisted tandem bicycle at a high cadence of 80-90 rpm with variable resistance. VE group: unassisted stationary bicycle at preferred cadence. All participants were instructed to maintain their heart rate within 60%-80% of their APMHR Study duration: 24 sessions (60 min) over 8 wk Assessment timepoints: wk 0, 8, 12 Outcome measures: UPDRS-III motor examination, rate of force production in bimanual task, estimated VO2 peak	Population: PD, mild to moderate Sample size: n=10 (8M,2F) FE group Sample size: n=5 Age: 58±2.1 y TSI/D: 7.9±7.0 y VE group Sample size: n=5 Age: 64±7.1 y TSI/D: 4.4±4.0 y	6	+	Forced leg cycling improved UE motor function (UPDRS increased 35% compared with baseline, *P*=.002), bimanual task performance (rate of force production increased 37%) and digit placement compared with self-paced cycling (no change, or slight decrease). UPDRS (mean±SD): baseline, wk 8, wk 12 FE: 48.4±11.3, 31.8±6.0 (*P*=.002, baseline), 46.0±13.6 VE: 49.0±13.8, 52.6±7.5, 55.5±7.2 Rigidity (mean±SD): baseline, wk 8, wk 12 FE: 11.8±3.2, 6.4±0.5, 9.8±1.9 VE: 9.8±2.9, 12.0±2.8, 12.8 ±3.1 Tremor (mean±SD): baseline, wk 8, wk 12 FE: 7.4±1.0, 4.6±1.0, 8.6±4.3 VE: 10.6±4.2, 10.2±3.4, 11.8±2.0 Bradykinesia (mean±SD): baseline, week 8, week 12 FE: 20.0±3.8, 14.6±4.2,19.8±4.1 VE: 20.0±5.0, 20.8±3.5, 22.8±5.4 CoP (mean±SD): baseline, week 8, week 12 FE (manipulating hand): 4.1 cm^2^, 1.1 mm^2^ (*P*<.01, baseline), 1.74 cm^2^ (*P*<.01, baseline) FE (stabilizing hand): 3.1 cm^2^, 1.0 mm^2^ (*P*<.01, baseline), 0.89 cm^2^ (*P*<.01, baseline) VE (manipulating hand): 3.8 cm^2^, 2.9 cm^2^, 2.9 cm^2^ VE (stabilizing hand): 3.1 cm^2^, 2.8 cm^2^, 2.5 cm^2^

Johannsen et al (2010)^36^	No	RCT: 2 parallel groups Training intervention: BATRAC versus BLETRAC Study duration: 10 sessions (30 min) over 5 wk Assessment timepoints: week 0, 6, and 18 Outcome measures: Primary: FMA UE and LE motor subscales. Secondary: 10MWT, TUG, STS, functional reach, postural sway, stabilization postperturbation, treadmill stride length, bimanual load lifting, and unilateral repetitive aiming tasks for the hand and foot	Population: stroke, mild BATRAC group Sample size: n=10 (7M,3F) Age: 68.1±10.2 y TSI/D: chronic; 51.1±31.6 mo BLETRAC group Sample size: n=11 (8M,3F) Age: 59.5±13.4 y TSI/D: chronic; 75.1±65.7 mo	7	˄	BLETRAC showed upper extremity improvements at wk 6 in hand aiming tasks. No improvements were maintained at the 3-mo follow-up. FMA UE (mean change [95% CI]): wk 6, wk 18 BLETRAC: 0.2 (−6.0 to 6.4), −4.8 (−9.4 to −0.2) BATRAC: 5.0 (−2 to 12), −3.9 (−7.1 to −0.7) Week 6 Group effect: U=42.0, *P*=.36; Week 18 Group effect: U=54.0, *P*=.94. Hand aiming (mean change [95% CI]): wk 6, wk 18 BLETRAC (paretic arm): 2.6 (1.3-3.8), −0.9 (−1.0 to 5.2) BATRAC (paretic arm): 0.9 (0.0-1.8), 0.6 (−0.4 to 1.6) BLETRAC (nonparetic arm): 4.3 (1.2-7.4), −1.6 (3.8-0.7) BATRAC (nonparetic arm): 2.1 (−1.0 to 5.2), −0.2 (−1.5 to 1.1) Week 6 Group effect: F_1,18_=2.15, *P*=.16, partial η^2^=.11 Side × group interaction: F_1,18_=0.11, *P*=.74, partial η^2^=.01. Week 18 Group effect: F_1,18_=3.38, *P*=.08, partial η2=.16 Side × group interaction: F_1,18_<0.01, *P*=.95, partial η^2^<.001)

Ridgel et al (2011)^37^	Yes	Non-RCT: 2 groups Training intervention: Cycling group: passive cycling, pedaling rates randomized to 60, 70, or 80 rpm; Cycling and Control group: watched one instructional video about the MOTOmed motorized cycle Study duration: 3 sessions (40 min) over 4 wk Assessment timepoints: before and immediately after each cycling bout Outcome measures: Kinesia device: kinematic data for UE tremor and bradykinesia of the more affected upper limb	Population: PD, mild to moderate Cycling group Sample size: n=20 (13M,7F) Age: 62.8±8.5 y TSI/D: 5.2±2.9 y Control group Sample size: n=12 (9M,3F) Age: 64.6±5.8 y TSI/D: 6.5±5.5 y	Not scored	+	Passive leg cycling reduced tremor and bradykinesia in the UE regardless of cycling rate. The cycling group showed a 0.25 decrease in tremor (affected side), and for bradykinesia, a 0.10 Hz increase in hand grasp frequency and 0.18 Hz increase in pronation/supination frequency at peak power. In contrast, no improvements were observed in the control group showing a 0.28 increase in tremor, 0.15 Hz decrease in hand grasp frequency, and a 0.19 Hz decrease in pronation/supination frequency at peak power. Group × time interaction effect: Tremor F_1,29_=4.385; *P*=.045; effect size, 0.13; observed power, 0.53; bradykinesia (hand grasp frequency) F_1,30_=10.15; *P*=.003; effect size, 0.25; observed power, 0.87; bradykinesia (pronation/supination frequency) F_1,30_=25.99; *P*=.000; effect size, 0.46; observed power, 0.87

Tyson et al (2015)^38^	No	RCT: 2 groups Training intervention: Leg group: fully supported ankles/knees/hips movements, joint movements against gravity, sitting, and standing. Mirror therapy group: finger/wrist/elbow movements, multiplanar joint movements against gravity, grasping, and moving objects Study duration: 20 sessions (30 min) over 4 wk Assessment timepoints: wk 0, 4, and 8 Outcome measures: Motricity Index, RASP, MAS (UE: biceps, LE: gastrocnemius), Star Cancellation test (neglect), BBT, grip strength, ARAT, Rivermead Mobility Index, Brunel Balance Assessment	Population: stroke, mixed severity Leg group Sample size: n=31 (23M,8F) Age: 65 (40-91) y TSI/D: early-subacute to chronic; 29 (7-133) d Mirror therapy group Sample size: n=62 (37M,25F) Age: 66 (26-88) y TSI/D: early-subacute to chronic; 18 (7-76) d	8	*=*	No UE improvements were observed in the leg exercise group (*P*>.05 for all). ARAT (mean±SD): baseline, wk 4, wk 8 Leg group: 10±15, 8.3±12.1, 8.8±13.8 Mirror therapy group: 13±18, 6.9±13.9, 8.5±14.5 Grip strength (mean±SD): baseline, wk 4, wk 8 Leg group: 2±3, 2.8±5.7, 3.6±5.4 Mirror therapy group: 4±7, 1.7±4.9, 2.3±6.8 Motricity Index (grip) (mean±SD): baseline, wk 4, wk 8 Leg group: 39±29, 6.8±16.8, 10.5±19.9 Mirror therapy group: 40±32, 9.1±18.3, 12.9±20.8 RASP (mean±SD): baseline, wk 4, wk 8 Leg group: 14±8, 2.4±6.2, 3.3±7.4 Mirror therapy group: 17±9, 0.3±7.7, 2.4±6.5 MAS (biceps) (mean±SD): baseline, wk 4, wk 8 Leg group: 1±1, -0.3±1.1, 0.1±1.6 Mirror therapy group: 1±1, -0.0±1.0, -0.1±1.1 BBT (mean±SD): baseline, wk 4, wk 8 Leg group: 4±9, 6±11.1, 7±12.3 Mirror therapy group: 5±9, 8.2±12.7, 9.3±14.9 Neglect (mean±SD): baseline, wk 4, wk 8 Leg group: 48±12, 2±7.4, 3.2±5.4 Mirror therapy group: 47±15, 4.4±10.8, 5.9±11.3

NOTE. "+" means a statistically significant or clinically relevant benefit/improvement in upper extremity (UE) outcome measures, “˄” means a nonsignificant improvement in UE outcome measures or no inferential statistics performed, and “=” means no statistically significant change in UE outcome measures.

Abbreviations: 2MWT, 2-Minute Walk Test; 6MWT, 6-Minute Walk Test; 10MWT, 10-Meter Walk Test; APMHR, age-predicted maximal heart rate; ARAT, Action Research Arm Test; ADL, activity of daily living; BATRAC, Bilateral Arm Training with Rhythmic Auditory Cueing; BBT, Box and Block Test; BLETRAC, Bilateral Leg Training with Rhythmic Auditory Cueing; BWSTT, body-weight-supported treadmill training; CoP, center of pressure; CSP, cortical silent period; FAC, Functional Ambulation Category; FDI, first dorsal interosseous; FE, forced exercise; FMA, Fugl-Meyer Assessment; IQR, interquartile range; JTHFT, Jebsen-Taylor Hand Function Test; LE, lower extremity; MAL, Motor Activity Log; MAS, Modified Ashworth Scale; MET, metabolic equivalent of task; MTS, Modified Tardieu Scale; N, Newton; PD, Parkinson disease; PEDro, Physiotherapy Evidence Database; PT, physical therapy; RASP, Rivermead Assessment of Sensory Perception; RCT, randomized control trial; ROM, range of motion; RPM, revolutions per minute; STS, Sit-to-Stand Test; TEMPA, Test Évaluant la Performance des Membres supérieurs des Personnes Âgées (Upper Extremity Performance Evaluation Test for the Elderly); TMS, transcranial magnetic stimulation; TSI/D, time since injury/diagnosis; TUG, timed Up-and-Go test; UE, upper extremity; UPDRS, Unified Parkinson Disease Rating Scales; VE, voluntary exercise; VO_2_, oxygen consumption; WMFT, Wolf Motor Function Test.

### Did leg training support arm and hand rehabilitation?

Half of the included studies reported a statistically significant improvement or clinically relevant benefit in upper extremity function after a leg-focused intervention, whereas the remaining studies showed no change. No study reported negative effects on upper extremity function. Only one study specifically hypothesized improved upper extremity outcomes after lower extremity intervention.^37^

### Randomized controlled trials

Kwakkel et al^28^ investigated the effects of arm and leg training intensities on functional recovery in 101 individuals with severe acute stroke. All participants received 20 weeks of usual care consisting of daily arm and leg rehabilitation (30 min), and ADL training (1.5 h/wk). Participants were randomly divided into an arm training (n=33), leg training (n=31), or control group (n=37), each receiving an additional 30 minutes (5/wk) of therapist-supervised sessions. Arm training consisted of leaning, punching a ball, grasping, and moving objects, and leg training consisted of sitting, standing, and weight-bearing exercises while standing and walking. The control group laid supine with the paretic limb passively immobilized by an inflatable pressure splint. An intention-to-treat analysis showed that additional arm training led to improvements in dexterity (Action Research Arm Test) of the paretic arm from week 12 onward (at wk 12: median [interquartile range] 3 [0-34] vs 0 [0-1] for controls, *P*<.05), compared with the control. In addition to significant improvements in walking performance, leg training resulted in improvements in upper extremity dexterity from week 6 onward (at wk 6: median 1 [0-43] vs 0 [0-1] for controls, *P*<.01), compared with the control (table 3). At the 26-week follow-up, both training groups performed better in the Action Research Arm Test compared with the controls (median 0 [0-2.25] for controls vs 4 [0-38], *P*<.05 for arm training; vs 3 [0-56], *P*<.01 for leg training), with no significant difference between training groups. The authors attributed the improvements in arm function for the leg group to arm usage during gait training and the higher proportion of leg group participants with greater baseline dexterity.^28^

Bourbonnais et al^30^ assessed strength and muscle activation after a 6-week progressive force-feedback training program (60 min, 3/wk) in 25 individuals with chronic moderate stroke. Twelve participants performed isometric training of the paretic lower limb with 16 torque combinations exerted at the hip and knee. Thirteen participants performed isometric training of the paretic upper limb with 16 torque combinations exerted at the shoulder, elbow, and forearm with/without handgrip exertion. Assessments included a 12-meter self-paced walking velocity test, a 2-minute walk test, a timed Up-and-Go test, Modified Ashworth Scale (MAS), Fugl-Meyer Assessment (FMA), upper extremity test for the elderly, box and block test, finger-to-nose test, and a pain assessment. Both the leg- and arm training groups demonstrated similar upper limb improvements for FMA (F=4.21, *P*=.021), finger-to-nose (F=4.43, *P*=.026), and box and block (F=3.86, *P*=.04) assessments immediately after the intervention and at week 14 (table 3). However, improvements did not translate into sustained functional gains. The authors postulated that this could be because of the lack of continued paretic limb use outside the study.^30^

Johannsen et al^36^ hypothesized that ten 30-minute sessions of bilateral leg training with rhythmic auditory cueing (BLETRAC) over five weeks would improve leg function and walking performance in chronic mild stroke. Twenty-one participants were randomly assigned to BLETRAC or a control group receiving bilateral arm training with rhythmic auditory cueing (BATRAC). Changes in FMA subscales served as their primary outcome, with a timed 10-Meter Walk Test (10MWT), treadmill walking parameters, and foot or hand aiming performance serving as secondary outcomes. No change was seen in either group for the FMA upper extremity subscale (0.2 [−6.0 to 6.4] vs 5.0 [−2 to 12], respectively; group main effect U=42.0, *P*=.36). Surprisingly, BLETRAC showed upper extremity improvements at week 6 in aiming tasks (mean increase of 2.6 for the paretic hand and 4.3 for the nonparetic hand), compared with BATRAC (mean increase 0.9 for the paretic hand and 2.1 for the nonparetic hand; group effect F_1,18_=2.15, *P*=.16) (table 3). The authors noted this could be because of the generalization of motor learning or compensatory trunk control strategies. No change from baseline or difference between groups was found at the 3-month follow-up.^36^

Fisher et al^34^ evaluated eight weeks of high (n=10) versus low-intensity (n=10) leg exercises, in comparison with no-exercise (control; n=10), in individuals with PD. High-intensity exercise included 24 sessions of progressive body-weight–supported treadmill training (BWSTT), defined in terms of metabolic equivalents (METs). The high-intensity group aimed for over 3 METs or 75% of maximum heart rate, whereas the low-intensity group aimed for under 3 METs or 50% of maximum heart rate for 45 minutes. The control group consisted of six 1-hour educational classes in addition to customary daily exercise routines across eight weeks. Changes in UPDRS and functional performance measures (10MWT, sit-to-stand test) were compared before and after. A subset of participants from each group took part in additional transcranial magnetic stimulation testing of corticomotor excitability. Interestingly, the high-intensity group showed lengthened cortical silent period duration in an intrinsic hand muscle (average 32 ms increase for the more affected hemisphere vs average 19.4 ms increase for the less affected hemisphere) (table 3), which is associated with reduced upper extremity PD symptoms, likely because of restoration of intracortical inhibition and facilitation.^39^ The low-intensity and no-exercise groups did not show the same benefits. Because of the preliminary nature of the trial, authors only reported descriptive analysis, no inferential statistics were performed. However, the authors suggest that high-intensity leg-focused exercise may induce neuroplasticity in upper extremity circuits by restoring normal motor processing.^34^

Ridgel et al^35^ focused on the effects of “forced exercise” in 10 individuals living with mild to moderate PD. Participants who performed leg cycling at their preferred “voluntary exercise” rate (VE; n=5) were compared with a “forced exercise” group (FE; n=5), in which the preferred voluntary rate was increased by 30% on an instructor-led tandem bicycle. Both groups exercised at similar aerobic intensities for 60 minutes (3/wk, 8 wk). Aerobic fitness, PD symptoms, motor function, and bimanual dexterity were assessed at week 0, 8, and 12. Forced leg cycling led to immediate improvements in rigidity (41%), tremor (38%), bradykinesia (28%), responsible for improved motor function (35% compared with baseline, *P*=.002) at week 8 (table 3), whereas no improvements were seen for the preferred cycling rate (*P*>.17), despite both groups showing similar improvements in aerobic fitness. Grip-load profile plots improved for both upper limbs, interlimb coordination assessed by grip time delay improved significantly (F_2,46_=4.634, *P*=.015), and the rate of grip force for the manipulating hand increased significantly (*P*=.006) for the FE group, with either no change (grip-load profiles, grip time delay) or a slight decrease (rate of grip force, *P*=.405) observed for the VE group. The FE group was significantly more consistent with digit placement of both stabilizing and manipulating limbs immediately after and at week 12 (*P*<.01 for both), as opposed to the VE group that did not exhibit any improvement. Notably, biomechanical measures of bimanual dexterity persisted for weeks after intervention. The authors speculated that forced exercise leads to a shift in central motor control processes and enhanced coordination of upper extremity functionality after a lower extremity intervention. One implication is that forced exercise may result in increased general neuroplasticity, which therefore attenuates PD symptoms.^35^

Dean et al^29^ compared a 4-week (60 min, 3/wk) supervised, progressive lower limb circuit training program (n=5) with upper limb training (control; n=4) for improved locomotor function in nine individuals living with mild chronic stroke. All assessments were performed for both groups and included a 6-Minute Walk Test, 10MWT, timed Up-and-Go test, sit-to-stand, step test, as well as grip strength and hand dexterity. The leg-focused group showed significant immediate (wk 4) and retained (2-mo follow-up) improvements in walking speed and endurance, force production, and balance when compared with the arm-focused group. Despite significant improvements in walking and balance outcomes in the leg-focused group, neither group showed significant improvements from baseline when assessing changes in grip strength and dexterity. The authors suggested that the failure to demonstrate improvement in the upper limbs could have been because of the high level of skill needed, lack of sensitivity in preselected assessments, or insufficient baseline level of hand function.^29^

Blennerhassett and Dite^31^ investigated the impact of task-oriented practice on motor recovery in 30 individuals during inpatient rehabilitation after moderate stroke. In addition to usual care, both groups received an additional hour of limb-specific exercises for four weeks (5/wk). The leg group performed endurance, mobility, balance, stretch, and strengthening exercises of the lower limb, whereas the arm group performed stretch and strengthening exercises, functional arm and hand tasks, and hand-eye coordination activities. Hand function, spasticity, and mobility performance were assessed before, immediately after the intervention, and at a 6-month follow-up. Although both groups improved significantly across all mobility assessments (leg group: *P<*.02; arm group *P*<.006), only the upper limb group made significant improvements in upper extremity assessments (Jebsen-Taylor Hand Function Test: *P=*.005; MAS upper arm: *P<*.001) (table 3).^31^

Pang et al^33^ assessed the effects of a community-based exercise program on paretic upper extremity function for 63 people with chronic stroke of varying severity. Participants were sex-matched and randomly divided into a therapist-supervised 19-week (60 min, 3/wk) arm or leg training group. Arm training consisted of progressive Theraband exercises, range of motion (ROM), weight-bearing activities, muscle strengthening, and functional activities. For participants unable to actively extend the wrist more than 20 degrees, 10-15 minutes of electrical muscle stimulation was performed on the wrist extensors. Progressive leg training consisted of walking, sitting, standing, alternate stepping, standing balance, partial squats, and toe raises. At week 19, the arm training group outperformed the leg group in the Wolf Motor Function Test of upper extremity function (functional ability score: between group, *P*=.003), and FMA (between group, *P*=.001) (table 3), whereas the leg training group showed little to no improvement in upper extremity outcomes. Importantly, the authors noted that their study was underpowered.^33^

Tyson et al^38^ conducted a multicenter trial with 94 participants with mixed stroke severity using self-directed exercises during acute inpatient stroke care. In addition to usual care, participants undertook 30 minutes of daily (5/wk, 4 wk) mirror therapy for the upper limb (n=63) or exercises for the lower limb (n=31). Mirror therapy concentrated on the fingers, wrist, and elbow and included progressive ROM exercises and functional activities such as reaching, grasping, and moving objects. Leg exercises concentrated on the ankles, knees, and hips and included progressive ROM and functional activities such as sitting and standing. Many outcomes in muscle strength, sensation, dexterity, spasticity, balance, and neglect were assessed at weeks 0, 4, and 8. Smaller nonsignificant improvements in neglect, upper limb strength, and dexterity were observed after leg exercises, with greater nonsignificant improvements in sensation and upper limb activity, as compared with mirror therapy group; however, group differences were minimal relative to data variability (all *P*>.05) (table 3). Lack of significance was attributed to the trial being underpowered with low reported adherence (totaling 5-15 min per session for 7 of the 28 d).^38^

### Nonrandomized controlled trial

Ridgel et al^37^ showed that acute bouts of passive leg cycling significantly improved arm tremor and bradykinesia in 20 individuals with mild to moderate PD. A motorized bicycle was programmed to passively rotate the participants’ legs at 60, 70, or 80 revolutions per minute, randomly, across three cycling sessions (40 min; 1 wk apart). A control group (n=12) was later recruited for a single session to watch a short instructional video about the bicycle. Functional assessments were carried out before and within 10 minutes of completing each session (passive cycling, video watching). The UPDRS-III motor evaluation and kinematic data were collected to evaluate tremors and bradykinesia in the most affected arm. The cycling group showed improvements in arm tremor (0.25 decrease) and bradykinesia scores (hand grasp frequency: 0.10Hz increase; pronation/supination frequency: 0.18Hz increase), regardless of cycling rate, opposed to worsening scores in the control group, as reflected by significant group and time interaction effects (tremor *P*=.045; hand grasp *P*=.003; pronation/supination *P*=.000) (table 3). The authors suggested that the acute improvements might be because of interlimb neural coupling and alterations in reflex pathways between arms and legs.^37^

### Case series

Hesse et al^27^ investigated the efficiency of treadmill training compared with traditional “Bobath” physical therapy (also known as neurodevelopmental treatment), in a crossover study in seven nonambulatory individuals after stroke. The study used an A-B-A design, where “A” comprised 30 minutes of partial BWSTT, rapidly reduced to full weight-bearing if possible, and “B” 45 minutes of Bobath therapy, each phase lasting three weeks (15 sessions). The Bobath method focused on gait performance, ADLs, and upper extremity motor function. Outcomes included gait performance, motor function, upper and lower extremity strength, and ankle and wrist tone, as assessed by the functional ambulation category, 10MWT, the Rivermead Motor Assessment, the Motricity Index, and MAS, respectively. No changes in upper limb strength or tone were observed in either group (table 3).^27^

Mehrholz et al^32^ evaluated the effects of a 6-week inpatient therapist-assisted jump training program (5-7 min; 5/wk) on measures of strength, spasticity, ROM, and gait in six individuals with hemiparesis and mild spasticity because of to stroke. The Motricity Index, FMA, modified Tardieu Scale, functional ambulation category, 10MWT, stride length, Rivermead Visual Gait Index, and 6-Minute Walk Test were measured at baseline and after six weeks. Despite significant improvement in multiple leg outcome measures, FMA remained unchanged over time for the upper limbs (39.5±2.1-39.2±2.0; *P*=.157) (table 3). The authors noted that aside from a small sample size, the lack of upper extremity improvements might be because of the assistance provided during the jump (holding one arm), whereas the affected arm was secured in a sling to prevent microtrauma of the glenohumeral joint.^32^

## Discussion

To our knowledge, this is the first scoping review attempting to identify studies that may reveal the influence of leg-focused interventions on arm and hand function for individuals living with neurologic conditions. Of the 12 identified studies, six demonstrated statistically significant or clinically relevant benefits of leg training on upper extremity outcome measures,^28^^,^^30^^,^^34^, ^35^, ^36^, ^37^ mostly as incidental findings. This reveals a dearth of inquiry into the potential for lower extremity training to facilitate upper extremity rehabilitation.

Research on quadruped animal models provides strong evidence for neural coupling between forelimb and hindlimb circuits during ambulation of all types.^8^^,^^40^, ^41^, ^42^ Humans retain several elements of this quadrupedal coupling, as seen in crawling,^43^ walking,^10^^,^^44^ running, cycling,^45^ and swimming.^10^ Disruption of these circuits leads to impaired coordination and altered reflex responses in both affected and unaffected limbs.^45^^,^^46^ Despite extensive research exploring the use of interlimb connectivity to facilitate recovery,^47^, ^48^, ^49^, ^50^, ^51^, ^52^ little emphasis has been placed on using legs to improve arm function.

### Interlimb coupling between legs

Studies in cats and rats have shown that isolated spinal networks are capable of generating rhythmic alternating flexion-extension movements in the hindlimbs even in the absence of supraspinal^53^^,^^54^ and forelimb input,^55^ demonstrating strong segmental coupling between the hindlimbs. In humans, leg-leg coupling is maintained during split-belt treadmill walking, where one leg moves at a different speed^56^, ^57^, ^58^ or direction.^58^^,^^59^ This complex coordination reflects a flexible interaction between supraspinal centers in the motor cortex, basal ganglia, cerebellum, spinal CPGs, peripheral sensory feedback, and descending supraspinal feedback loops that optimize leg movement during walking and other activities, even under extreme circumstances.^10^^,^^45^

### Interlimb coupling between arms

Studies with cats demonstrate that bilateral coordination of the forelimbs during locomotion is driven by cervical CPGs, similar to those controlling the hindlimbs.^11^^,^^60^^,^^61^ As with quadrupeds, it is believed that humans have a distinct cervical CPG mediating upper limb coupling. Arm-arm coupling is evident during rhythmic tasks, such as arm swing during walking and arm cycling, where phase-dependent cervical spinal cord network modulation enables coordination between the arms.^62^^,^^63^ Other tasks requiring bilateral power gripping—such as opening a bottle or lifting something heavy—involve coordinated neural coupling for force production and movement control.^64^ However, arm-arm coupling is weaker than leg-leg coupling because of the greater demand for independent and versatile movements such as piano playing or fine object manipulation.^12^^,^^45^^,^^62^^,^^64^, ^65^, ^66^

### Interlimb coupling between arms and legs

Animal studies have shown that coordinated rhythmic movements of forelimbs and hindlimbs are controlled by distinct yet interconnected spinal networks that can operate autonomously from each other or supraspinal input.^8^^,^^53^^,^^67^, ^68^, ^69^ In humans, arm-leg neural coupling is most evident during walking and running, where arm swing and leg movement are tightly coordinated.^10^^,^^45^^,^^70^ Although locomotion can be achieved without propulsive arm activity, natural rhythmic patterns still occur,^12^ even when arms are restrained,^71^ or with varying perturbations on a split-belt treadmill,^72^ and is strengthened as walking velocity increases.^73^

Arm-leg coupling is also evident during nonwalking tasks such as arm-leg cycling and recumbent stepping, where active arm movement modulates leg reflexes and enhances neural activation and recruitment,^15^^,^^74^^,^^75^ indicating bidirectional neural pathways for any type of rhythmic limb movement.^18^^,^^63^ In fact, rhythmic hand-walking on an overhead treadmill while side-lying can induce locomotor-like activity in the legs.^76^ Furthermore, reflexes in the arms can be modulated by rhythmic leg cycling,^65^ and evoked by stimulation at the ankle.^18^ Overall, these studies show patterns of preprogrammed rhythmic interlimb movements in line with the claim that human gait is still a form of quadrupedal locomotion^10^ with bidirectional influence between arm and leg CPGs.

### Interlimb coupling in neurologic disorders

Our scoping review supports the potential to leverage leg-focused training to improve upper limb function in people with neurologic disorders. However, exploiting this potential will require better understanding of interlimb coupling as a target for enhancing neurorehabilitation. In neurologic conditions such as SCI, rehabilitative strategies that engage both arms and legs have shown potential in re-establishing interlimb coupling and improving motor function.^45^ For instance, arm swing during walking promotes more normal electromyographic patterns in leg muscles of individuals with incomplete cervical SCI.^20^ Despite some poststroke hemiparesis studies showing exacerbated deficits in paretic leg coordination during nonparetic leg activity,^77^ other studies have shown evidence of effective task transfer to impaired limbs, with improved neurologic function and walking performance across different interlimb training modalities.^78^, ^79^, ^80^

With regard to individuals after a stroke, Kwakkel et al,^28^ Bourbonnais et al,^30^ and Johannsen et al^36^ each showed that leg-focused rehabilitation improved upper limb performance to a greater degree than control interventions. In PD, Ridgel et al^35^^,^^37^ demonstrated that 12 sessions of “forced” tandem-cycling and passive cycling at different training rates mitigated PD symptoms of upper extremity bradykinesia and tremors. Likewise, Fisher et al^34^ observed increased corticomotor excitability projecting toward intrinsic hand muscles in addition to improved walking performance in individuals with PD undergoing high-intensity BWSTT (45 min; 3/wk; 8 wk), in comparison with low-intensity BWSTT or nil.

These studies provide evidence of bidirectional coupling and suggest that leg movements may facilitate motor recovery in the arms by enhancing overall neural plasticity. Although these studies hint at potential leg training benefits on arm function, not all studies showed improvement, and none specifically aimed to use leg training to improve upper extremity function. Factors such as study design, small sample size, poor adherence, training volume and intensity, sensitivity of upper limb assessments, as well as inclusion and exclusion criteria, could have impacted these results. Further research is needed to elucidate the role of leg exercises in facilitating arm and hand function across different neurologic conditions.

### Study limitations

The purpose of the scoping review was to summarize the existing literature on an unexplored question rather than to definitively answer it. This review used a comprehensive search strategy across five primary databases, nine gray literature databases, and secondary references to ensure relevant studies were included. Systematic source handling and selection criteria were used to maintain objectivity. The findings highlight an underexplored topic, specifically leg-focused interventions for improving upper extremity outcomes, which warrants greater critical appraisal.

However, the lack of studies on this topic limits the strength of conclusions. Only 12 relevant peer-reviewed articles were identified across 77 years, and their methodological quality ranged from 5 to 8 on the Physiotherapy Evidence Database scale, with a score of 8 considered optimal for evaluating complex interventions such as exercise.^26^ Included studies had heterogeneous designs, interventions, and outcomes. Many other studies were excluded because of unclear interventions, no leg-only training focus, or a lack of detailed upper extremity outcome measurements. No risk of bias assessment was conducted, limiting the strength of evidence. Furthermore, the review was open to any neurologic disorder; however, the identified studies were highly focused on stroke survivors and individuals living with PD, limiting generalizability to other neurologic conditions such as SCI.

Data revealing the potential influence of leg training on arm function were often an ancillary finding. Only six of the 12 studies reported improvements in upper extremity function, whereas the others reported no change. Considerable heterogeneity in study design, exercise parameters, outcome measures, and patient populations, along with small sample sizes, likely contributed to inconsistent findings, making it difficult to compare. Most studies lacked objective neurophysiology or radiological assessments (eg, neuroimaging, transcranial magnetic stimulation, electromyography), which are critical for assessing changes related to neural plasticity and interlimb coupling. Further understanding of how the stage of recovery, severity, and condition progression, as well as the duration and specific types of interventions, can influence the observed outcomes will be essential to interpret the findings and improve the design of subsequent trials.

### Implications for further research

This review found evidence that leg training has the potential to improve arm and hand function in people with neurologic conditions. However, the extremely limited and heterogeneous nature of the results highlights significant gaps in our understanding of interlimb coupling from legs to arms as an innovative rehabilitation strategy. Further exploration of this approach is warranted not only in stroke and PD but also in other neurologic conditions, such as SCI, ALS, multiple sclerosis, and cerebral palsy.

## Conclusions

This review highlights a critical gap in understanding the therapeutic impact of leg exercises on arm function and rehabilitation. Further research is needed to explore how various leg training modes, intensity, and frequency influence arm rehabilitation. Expanding this evidence with more objective measures could support integrating interlimb connectivity into routine neurorehabilitation for individuals living with neurologic conditions.

## Disclosure

The investigators have no financial or nonfinancial disclosures to make in relation to this project.

## Appendix Supplementary materials

Image, application 1

## Footnotes

Supplementary material associated with this article can be found, in the online version, at doi:10.1016/j.arrct.2025.100572.

## References

[1] Sherrington CS. (1910). Flexion-reflex of the limb, crossed extension-reflex, and reflex stepping and standing. J Physiol.

[2] Brown TG. (1914). On the nature of the fundamental activity of the nervous centres; together with an analysis of the conditioning of rhythmic activity in progression, and a theory of the evolution of function in the nervous system. J Physiol.

[3] Dimitrijevic M.R., Gerasimenko Y., Pinter MM. (1998). Evidence for a spinal central pattern generator in humans. Ann N Y Acad Sci.

[4] Grillner S., Terjung R. (1981). Comprehensive physiology.

[5] Hultborn H., Nielsen JB. (2007). Spinal control of locomotion – from cat to man. Acta Physiol.

[6] Rossignol S., Dubuc R., Gossard JP. (2006). Dynamic sensorimotor interactions in locomotion. Physiol Rev.

[7] Stein PSG. (2018). Central pattern generators in the turtle spinal cord: selection among the forms of motor behaviors. J Neurophysiol.

[8] Frigon A. (2017). The neural control of interlimb coordination during mammalian locomotion. J Neurophysiol.

[9] Côté M.P., Murray L.M., Knikou M. (2018). Spinal control of locomotion: individual neurons, their circuits and functions. Front Physiol.

[10] Dietz V. (2002). Do human bipeds use quadrupedal coordination?. Trends Neurosci.

[11] Miller S., Van Der Burg J., Van Der Meché F. (1975). Coordination of movements of the kindlimbs and forelimbs in different forms of locomotion in normal and decerebrate cats. Brain Res.

[12] Zehr E.P., Duysens J. (2004). Regulation of arm and leg movement during human locomotion. Neuroscientist.

[13] McIlroy W.E., Maki BE. (1995). Early activation of arm muscles follows external perturbation of upright stance. Neurosci Lett.

[14] Marigold D.S., Patla AE. (2002). Strategies for dynamic stability during locomotion on a slippery surface: effects of prior experience and knowledge. J Neurophysiol.

[15] Huang H.J., Ferris DP. (2004). Neural coupling between upper and lower limbs during recumbent stepping. J Appl Physiol.

[16] Huang H.J., Ferris DP. (2009). Upper and lower limb muscle activation is bidirectionally and ipsilaterally coupled. Med Sci Sports Exerc.

[17] Fernandez-Ballesteros M.L., Buchthal F., Rosenfalck P. (1965). The pattern of muscular activity during the arm swing of natural walking. Acta Physiol Scand.

[18] Haridas C., Zehr EP. (2003). Coordinated interlimb compensatory responses to electrical stimulation of cutaneous nerves in the hand and foot during walking. J Neurophysiol.

[19] Weersink J.B., de Jong B.M., Maurits NM. (2022). Neural coupling between upper and lower limb muscles in Parkinsonian gait. Clin Neurophysiol.

[20] Kawashima N., Nozaki D., Abe M.O., Nakazawa K. (2008). Shaping appropriate locomotive motor output through interlimb neural pathway within spinal cord in humans. J Neurophysiol.

[21] Hubli M., Dietz V. (2013). The physiological basis of neurorehabilitation–locomotor training after spinal cord injury. J Neuroengineering Rehabil.

[22] Kuo H.I., Hsieh M.H., Lin Y.T., Kuo M.F., Nitsche MA. (2023). A single bout of aerobic exercise modulates motor learning performance and cortical excitability in humans. Int J Clin Health Psychol.

[23] Peters M.D., Godfrey C., McInerney P., Munn Z., Tricco A.C., Khalil K. (2024). JBI Manual for Evidence Synthesis.

[24] Tricco A.C., Lillie E., Zarin W. (2018). PRISMA Extension for Scoping Reviews (PRISMA-ScR): checklist and explanation. Ann Intern Med.

[25] Covidence systematic review software, 2021, Veritas Health Innovation, Melbourne, Australia. Available at: https://covidence.org/. Accessed November 18, 2025.

[26] PEDro scale. 1999. Available at: https://pedro.org.au/english/resources/pedro-scale/. Accessed November 28, 2025.

[27] Hesse S., Bertelt C., Jahnke M.T. (1995). Treadmill training with partial body weight support compared with physiotherapy in nonambulatory hemiparetic patients. Stroke.

[28] Kwakkel G., Wagenaar R.C., Twisk J.W., Lankhorst G.J., Koetsier JC. (1999). Intensity of leg and arm training after primary middle-cerebral-artery stroke: a randomised trial. Lancet.

[29] Dean C.M., Richards C.L., Malouin F. (2000). Task-related circuit training improves performance of locomotor tasks in chronic stroke: a randomized, controlled pilot trial. Arch Phys Med Rehabil.

[30] Bourbonnais D., Bilodeau S., Lepage Y., Beaudoin N., Gravel D., Forget R. (2002). Effect of force-feedback treatments in patients with chronic motor deficits after a stroke. Am J Phys Med Rehabil.

[31] Blennerhassett J., Dite W. (2004). Additional task-related practice improves mobility and upper limb function early after stroke: a randomised controlled trial. Aust J Physiother.

[32] Mehrholz J., Rutte K., Pohl M. (2006). Jump training is feasible for nearly ambulatory patients after stroke. Clin Rehabil.

[33] Pang M.Y., Harris J.E., Eng JJ. (2006). A Community-based upper-extremity group exercise program improves motor function and performance of functional activities in chronic stroke: a randomized controlled trial. Arch Phys Med Rehabil.

[34] Fisher B.E., Wu A.D., Salem G.J. (2008). The effect of exercise training in improving motor performance and corticomotor excitability in people with early Parkinson’s disease. Arch Phys Med Rehabil.

[35] Ridgel A.L., Vitek J.L., Alberts JL. (2009). Forced, not voluntary, exercise improves motor function in Parkinson’s disease patients. Neurorehabil Neural Repair.

[36] Johannsen L., Wing A.M., Pelton T. (2010). Seated bilateral leg exercise effects on hemiparetic lower extremity function in chronic stroke. Neurorehabil Neural Repair.

[37] Ridgel A.L., Muller M.D., Kim C.H., Fickes E.J., Mera TO. (2011). Acute effects of passive leg cycling on upper extremity tremor and Bradykinesia in Parkinson’s disease. Phys Sportsmed.

[38] Tyson S., Wilkinson J., Thomas N. (2015). Phase II pragmatic randomized controlled trial of patient-led therapies (Mirror Therapy and Lower-Limb Exercises) during inpatient stroke rehabilitation. Neurorehabil Neural Repair.

[39] Lefaucheur JP. (2005). Motor cortex dysfunction revealed by cortical excitability studies in Parkinson’s disease: influence of antiparkinsonian treatment and cortical stimulation. Clin Neurophysiol.

[40] Aoi S., Manoonpong P., Ambe Y., Matsuno F., Wörgötter F. (2017). Adaptive control strategies for interlimb coordination in legged robots: a review. Front Neurorobotics.

[41] Grillner S. (1975). Locomotion in vertebrates: central mechanisms and reflex interaction. Physiol Rev.

[42] Grillner S. (1985). Neurobiological bases of rhythmic motor acts in vertebrates. Science.

[43] Righetti L., Nylén A., Rosander K., Ijspeert AJ. (2015). Kinematic and gait similarities between crawling human infants and other quadruped mammals. Front Neurol.

[44] Dietz V., Michel J. (2009). Human bipeds use quadrupedal coordination during locomotion. Ann N Y Acad Sci.

[45] Zehr E.P., Barss T.S., Dragert K. (2016). Neuromechanical interactions between the limbs during human locomotion: an evolutionary perspective with translation to rehabilitation. Exp Brain Res.

[46] Dietz V. (2011). Quadrupedal coordination of bipedal gait: implications for movement disorders. J Neurol.

[47] Farthing J.P., Zehr EP. (2014). Restoring symmetry: clinical applications of cross-education. Exerc Sport Sci Rev.

[48] Ruddy K.L., Carson RG. (2013). Neural pathways mediating cross education of motor function. Front Hum Neurosci.

[49] Kawakita H., Kameyama O., Ogawa R., Hayes K.C., Wolfe D.L., Allatt RD. (1991). Reinforcement of motor evoked potentials by remote muscle contraction. J Electromyogr Kinesiol.

[50] Sasaki A., Kaneko N., Masugi Y., Milosevic M., Nakazawa K. (2020). Interlimb neural interactions in corticospinal and spinal reflex circuits during preparation and execution of isometric elbow flexion. J Neurophysiol.

[51] Tazoe T., Sakamoto M., Nakajima T., Endoh T., Komiyama T. (2007). Effects of remote muscle contraction on transcranial magnetic stimulation-induced motor evoked potentials and silent periods in humans. Clin Neurophysiol.

[52] Komeilipoor N., Ilmoniemi R.J., Tiippana K., Vainio M., Tiainen M., Vainio L. (2017). Preparation and execution of teeth clenching and foot muscle contraction influence on corticospinal hand-muscle excitability. Sci Rep.

[53] Forssberg H., Grillner S., Halbertsma J. (1980). The locomotion of the low spinal cat. I. Coordination within a hindlimb. Acta Physiol Scand.

[54] Kiehn O., Kjaerulff O. (1998). Distribution of central pattern generators for rhythmic motor outputs in the spinal cord of limbed vertebrates. Ann N Y Acad Sci.

[55] Harnie J., Audet J., Mari S. (2022). State- and condition-dependent modulation of the hindlimb locomotor pattern in intact and spinal cats across speeds. Front Syst Neurosci.

[56] Dietz V., Zijlstra W., Duysens J. (1994). Human neuronal interlimb coordination during split-belt locomotion. Exp Brain Res.

[57] Prokop T., Berger W., Zijlstra W., Dietz V. (1995). Adaptational and learning processes during human split-belt locomotion: interaction between central mechanisms and afferent input. Exp Brain Res.

[58] Yang J.F., Lamont E.V., Pang MYC. (2005). Split-belt treadmill stepping in infants suggests autonomous pattern generators for the left and right leg in humans. J Neurosci.

[59] Choi J.T., Bastian AJ. (2007). Adaptation reveals independent control networks for human walking. Nat Neurosci.

[60] Drew T., Rossignol S. (1987). A kinematic and electromyographic study of cutaneous reflexes evoked from the forelimb of unrestrained walking cats. J Neurophysiol.

[61] Yamaguchi T. (1992). Activity of cervical neurons during forelimb fictive locomotion in decerebrate cats. Jpn J Physiol.

[62] Zehr E.P., Collins D.F., Frigon A., Hoogenboom N. (2003). Neural control of rhythmic human arm movement: phase dependence and task modulation of hoffmann reflexes in forearm muscles. J Neurophysiol.

[63] Zehr E.P., Kido A. (2001). Neural control of rhythmic, cyclical human arm movement: task dependency, nerve specificity and phase modulation of cutaneous reflexes. J Physiol.

[64] Dietz V. (2021). Neural coordination of bilateral power and precision finger movements. Eur J Neurosci.

[65] Carroll T.J., Zehr E.P., Collins DF. (2005). Modulation of cutaneous reflexes in human upper limb muscles during arm cycling is independent of activity in the contralateral arm. Exp Brain Res.

[66] Porter R. (1985). The corticomotoneuronal component of the pyramidal tract: corticomotoneuronal connections and functions in primates. Brain Res.

[67] Edgerton V.R., Roy R.R., Hodgson J.A., Prober R.J., de Guzman C.P., de Leon R. (1992). Potential of adult mammalian lumbosacral spinal cord to execute and acquire improved locomotion in the absence of supraspinal input. J Neurotrauma.

[68] Forssberg H., Grillner S., Halbertsma J., Rossignol S. (1980). The locomotion of the low spinal cat. II. Interlimb coordination. Acta Physiol Scand.

[69] Leblond H., L’Esperance M., Orsal D., Rossignol S. (2003). Treadmill locomotion in the intact and spinal mouse. J Neurosci.

[70] Zehr E.P., Hundza S.R., Vasudevan EV. (2009). The quadrupedal nature of human bipedal locomotion. Exerc Sport Sci Rev.

[71] Kuhtz-Buschbeck J.P., Jing B. (2012). Activity of upper limb muscles during human walking. J Electromyogr Kinesiol.

[72] MacLellan M.J., Qaderdan K., Koehestanie P., Duysens J., McFadyen BJ. (2013). Arm movements during split-belt walking reveal predominant patterns of interlimb coupling. Hum Mov Sci.

[73] Donker S.F., Beek P.J., Wagenaar R.C., Mulder T. (2001). Coordination between arm and leg movements during locomotion. J Mot Behav.

[74] Frigon A., Collins D.F., Zehr EP. (2004). Effect of rhythmic arm movement on reflexes in the legs: modulation of soleus H-reflexes and somatosensory conditioning. J Neurophysiol.

[75] Sakamoto M., Tazoe T., Nakajima T., Endoh T., Shiozawa S., Komiyama T. (2007). Voluntary changes in leg cadence modulate arm cadence during simultaneous arm and leg cycling. Exp Brain Res.

[76] Sylos-Labini F., Ivanenko Y.P., Maclellan M.J., Cappellini G., Poppele R.E., Lacquaniti F. (2014). Locomotor-like leg movements evoked by rhythmic arm movements in humans. PloS One.

[77] Kautz S.A., Patten C. (2005). Interlimb influences on paretic leg function in poststroke hemiparesis. J Neurophysiol.

[78] Arya K.N., Pandian S., Kumar V. (2019). Effect of activity-based mirror therapy on lower limb motor-recovery and gait in stroke: a randomised controlled trial. Neuropsychol Rehabil.

[79] Kaupp C., Pearcey G.E.P., Klarner T. (2018). Rhythmic arm cycling training improves walking and neurophysiological integrity in chronic stroke: the arms can give legs a helping hand in rehabilitation. J Neurophysiol.

[80] Klarner T., Barss T.S., Sun Y., Kaupp C., Loadman P.M., Zehr EP. (2016). Exploiting interlimb arm and leg connections for walking rehabilitation: a training intervention in stroke. Neural Plast.

