# Supplementary material for: Do Leg-Focused Exercises Improve Arm and Hand Function for Individuals With Neurologic Disorders? A Scoping Review
Source: Arch Rehabil Res Clin Transl. 2026 Jan 21;8(2):100572. doi: 10.1016/j.arrct.2025.100572 (PMC13282757; doi:10.1016/j.arrct.2025.100572)
Supplement: Supplementary file 1 [file mmc1.docx]

**Supplemental material**

Supplementary Table S1

Table 2. **Search strategy - expanded.**

| 1. **Peer-reviewed sources continued:** PubMed, EMBASE (Ovid), Cumulative Index to Nursing and Allied Health Literature (CINAHL), Scopus, and Cochrane CENTRAL.  **Return date range**: 1/1/1947 to 10/28/2025.  **PubMed Query [1,102 results]**  Utilizing proximity/adjacency search between 4-8 keywords and unlimited truncation of selective words in a citation's title, collection title, abstract, other abstract, and author keywords using the [tiab] tag and asterisk.  Modified search: ((leg[tiab] OR "leg exercise"[Title/Abstract:~5] OR "leg cycling"[Title/Abstract:~5] OR "leg walking"[Title/Abstract:~8] OR "leg running"[Title/Abstract:~5] OR "leg movement"[Title/Abstract:~5] OR "leg sport"[Title/Abstract:~5] OR "leg strength"[Title/Abstract:~5] OR "leg activity"[Title/Abstract:~5])) AND (((Neurological Rehabilitation) OR "Nervous System Diseases"[Mesh] OR "spinal injuries"[Title/Abstract:~5] OR "spinal injury"[Title/Abstract:~5] OR neurolog*[tiab] OR spinal[tiab]) AND ((hand[tiab] OR "hand strength"[Title/Abstract:~5] OR arm[tiab] OR "upper extremity"[Title/Abstract:~4] OR "upper limbs"[Title/Abstract:~4]) AND ((Function[tiab] OR Recover*[tiab] OR Strength[tiab] OR Dexter*[tiab] OR Grip[tiab] OR "Recovery of Function"[Mesh]))))  **EMBASE (Ovid) Query [181 results]**  Utilizing proximity/adjacency search between 4-8 keywords/free-text and unlimited truncation of selective words in a citation's title, collection title, abstract, other abstract, and author keywords using the [ab,ti, tw] tag and asterisk.  Embase Classic+Embase  1      exp hand function/  *2      hand.ab,ti,tw.*  *3      (hand adj5 strength).ab,ti,tw.*  *4      arm.ab,ti,tw.*  *5      (upper adj4 extremity).ab,ti,tw.*  *6      (upper adj4 limbs).ab,ti,tw.*  *7      (Function or Recover* or Strength or Dexter* or Grip).ab,ti,tw.*  *8      2 or 3 or 4 or 5 or 6*  *9      7 and 8*  10    1 or 9  *11    (neurolog* or spinal).ab,ti,tw.*  *12    (spinal adj5 injuries).ab,ti,tw.*  *13    (spinal adj5 injury).ab,ti,tw.*  14    exp neurologic disease/  15    11 or 12 or 13 or 14  *16    (leg adj5 exercise).ab,ti,tw.*  *17    (leg adj5 cycling).ab,ti,tw.*  *18    (leg adj8 walking).ab,ti,tw.*  *19    (leg adj5 running).ab,ti,tw.*  *20    (leg adj5 movement).ab,ti,tw.*  *21    (leg adj5 sport).ab,ti,tw.*  *22    (leg adj5 strength).ab,ti,tw.*  *23    (leg adj5 activity).ab,ti,tw.*  24    exp leg/ or exp leg movement/  25    leg.ab,ti,tw.  26    16 or 17 or 18 or 19 or 20 or 21 or 22 or 23 or 24 or 25  27    10 and 15 and 26  *28    11 or 12 or 13*  *29    16 or 17 or 18 or 19 or 20 or 21 or 22 or 23*  *30    9 and 28 and 29*  **CINAHL Query [541 results]**  Utilizing proximity/adjacency search between 4-5 words and unlimited truncation of selective words in a citation's title, abstract, and subject heading fields using the [AB][TI] tags and asterisk.  #      Query  Limiters/Expanders  *S15  S7 AND S11 AND S14*  *S14  S12 OR S13*  *S13  TI leg OR AB leg*  *S12  TI (leg N5 (exercise OR walking OR cycling OR running OR movement OR sport OR strength OR activity)) OR AB (leg N5 (exercise OR walking OR cycling OR running OR movement OR sport OR strength OR activity))*  *S11  S8 OR S9 OR S10*  *S10  (MH "Nervous System Diseases+")*  *S9    TI (neurolog* OR spinal) OR AB (neurolog* OR spinal)*  *S8    TI spinal N5 injuries OR AB spinal N5 injuries*  *S7    S5 AND S6*  *S6    TI (Function OR Recover* OR Strength OR Dexter* OR Grip) OR AB (Function OR Recover* OR Strength OR Dexter* OR Grip)*  *S5    S1 OR S2 OR S3 OR S4*  *S4    TI (hand OR arm) OR AB (hand OR arm)*  *S3    TI upper N4 limbs OR AB upper N4 limbs*  *S2    TI upper N4 extremity OR AB upper N4 extremity*  *S1    TI hand N5 strength OR AB hand N5 strength*  **Scopus Query [682 results]**  Utilizing proximity/adjacency search between 3-5 words and unlimited truncation of selective words in a citation's title, abstract, and subject heading fields using the [TITLE-ABS] tag and asterisk.    Modified search: ((TITLE-ABS (leg W/5 (exercise OR cycling OR walking OR running OR movement OR sport OR strength OR activity))) OR (TITLE-ABS (leg W/8 walking)) OR (TITLE-ABS (leg OR legs ))) AND ((TITLE-ABS (spinal W/5 injuries OR spinal W/5 injury)) OR (TITLE-ABS (neurolog* OR spinal)) OR (TITLE-ABS (nervous W/3 (disease* OR disorder*)))) AND ((TITLE-ABS (function OR recover* OR strength OR dexter* OR grip)) AND ((TITLE-ABS (hand OR arm)) OR (TITLE-ABS (hand W/5 strength OR upper W/4 extremity OR upper W/4 limbs))))    **Cochrane CENTRAL Register of Controlled Trials Query [292 results]**  Utilizing proximity/adjacency search between 6 words and unlimited truncation of selective words in a citation's title, abstract, and keywords.  Modified search: function OR recover* OR strength OR dexter* OR grip in Title Abstract Keyword AND hand OR arm OR hand NEAR strength OR upper NEAR extremity OR upper NEAR limbs in Title Abstract Keyword AND spinal NEAR injury* OR neurolog* OR spinal OR (nervous NEAR disease* OR disorder*) in Title Abstract Keyword AND leg NEAR (exercise OR cycling OR walking OR running OR movement OR sport OR strength OR activity) in Title Abstract |
| --- |
| 2. **Grey literature sources:** Google Scholar, medRxiv, Australia New Zealand Clinical Trials Registry (ANZCTR), European Union Clinical Trials Registry (EU-CTR), US government web-based resource ClinicalTrials.gov, World Health Organization (WHO) Library, WHO Global Index Medicus (WHO-GIM), and WHO International Clinical Trials Registry Platform (WHO-ICTRP), ProQuest Dissertation/Theses.    **Return date range**: 1/1/1947 to 11/29/2023.  **Google Scholar Search Query [first 5 pages (50 results of 8010 results)]**  Modified search: intitle:(Function\|recover*\|strength\|dexter*\|grip)(hand\|arm\|hand AROUND (4) strength\|upper AROUND (4) extremity\|upper AROUND (4) limbs)(spinal AROUND (5) injuries\|spinal AROUND (5) injury\|neurolog* OR spinal)(leg AROUND (5) exercise OR cycling OR walking OR running OR movement OR sport OR strength OR activity)  **MedXRiv Query [11 results]**  Modified search: (leg OR "leg exercise" OR "leg cycling" OR "leg walking" OR "leg running" OR "leg movement" OR "leg sport" OR "leg strength" OR "leg activity") (Neurological Rehabilitation OR "Nervous System Diseases" OR "spinal injuries" OR "spinal injury" OR neurolog* OR spinal) ((hand OR "hand strength" OR arm OR "upper extremity" OR "upper limbs") (Function OR Recover* OR Strength OR Dexter* OR Grip OR "Recovery of Function"))  **ANZCTR Query [35 results]**  Modified search: hand AND (function OR strength) AND (spinal OR neuro*) AND (injur* OR rehab*)  **EU-CTR Query [6 results]**  Modified search: hand AND (function OR strength) AND (spinal OR neuro*) AND (injur* OR rehab*)  **ClinicalTrials.gov Query [12 results]**  Modified search: (Function\|recover*\|strength\|dexter\|grip)(hand\|arm\|"hand strength"\|"upper extremity"\|"upper limbs")("spinal injuries"\|neurolog*\|spinal)(leg exercise\|cycling\|walking\|running\|movement\|sport\|strength\|activity)  **WHO Library Query [8 results]**  Modified search: hand AND (function OR strength) AND (spinal OR neuro*) AND (injur* OR rehab*)  **WHO-GIM Query [20 results]**  Modified search: (leg OR "leg exercise" OR "leg cycling" OR "leg walking" OR "leg running" OR "leg movement" OR "leg sport" OR "leg strength" OR "leg activity") (neurological rehabilitation OR "Nervous System Diseases" OR "spinal injuries" OR "spinal injury" OR neurolog* OR spinal) ((hand OR "hand strength" OR arm OR "upper extremity" OR "upper limbs") (function OR recover* OR strength OR dexter* OR grip OR "Recovery of Function"))  **WHO-ICTRP Query [16 results]**  Modified search: (leg AND (exercise OR cycling OR walking OR running OR movement OR sport OR strength OR activity)) AND (Neurological Rehabilitation OR "Nervous System Diseases" OR "spinal injuries" OR "spinal injury" OR neurolog* OR spinal) AND ((hand OR "hand strength" OR arm OR "upper extremity" OR "upper limbs") AND (Function OR Recover* OR Strength OR Dexter* OR Grip OR "Recovery of Function"))  **ProQuest Dissertation & Theses Query [43 results]**  Modified search: title((leg AND (exercise OR cycling OR walking OR running OR movement OR sport OR strength OR activity)) AND (Neurological Rehabilitation OR "Nervous System Diseases" OR "spinal injuries" OR "spinal injury" OR neurolog* OR spinal) AND ((hand OR "hand strength" OR arm OR "upper extremity" OR "upper limbs") AND (Function OR Recover* OR Strength OR Dexter* OR Grip OR "Recovery of Function"))) OR abstract((leg AND (exercise OR cycling OR walking OR running OR movement OR sport OR strength OR activity)) AND (Neurological Rehabilitation OR "Nervous System Diseases" OR "spinal injuries" OR "spinal injury" OR neurolog* OR spinal) AND ((hand OR "hand strength" OR arm OR "upper extremity" OR "upper limbs") AND (Function OR Recover* OR Strength OR Dexter* OR Grip OR "Recovery of Function"))) |
| 3. **Secondary references:** additional references were sought by examining the citations of included articles. |
